# Crosstalk between cancer stem cells and the tumor microenvironment drives progression of premalignant oral epithelium

**DOI:** 10.3389/froh.2022.1095842

**Published:** 2023-01-10

**Authors:** Peter J. Polverini, Felipe Nör, Jacques E. Nör

**Affiliations:** ^1^ Department of Periodontics and Oral Medicine, University of Michigan School of Dentistry, Ann Arbor, MI, United States; ^2^ Department of Cariology, Restorative Sciences, and Endodontics, University of Michigan School of Dentistry, Ann Arbor, MI, United States

**Keywords:** angiogenesis, cancer stem cells, carcinogenesis, endothelial cells, epithelial dysplasia, macrophages, premalignant, tumor progression

## Abstract

Cancer stem cells (CSC) are a subpopulation of cancer cells that exhibit properties of self-renewal and differentiation and have been implicated in metastasis and treatment failures. There is mounting evidence that carcinogen-initiated mucosal epithelial stem cells acquire the CSC phenotype following exposure to environmental or infectious mutagens and are responsible for promoting the malignant transformation of premalignant (dysplastic) epithelium. CSC further contribute to the progression of dysplasia by activating signaling pathways through crosstalk with various cell populations in the tumor microenvironment. Two cell types, tumor-associated macrophages (TAM) and vascular endothelial cells (EC) nurture CSC development, support CSC stemness, and contribute to tumor progression. Despite mounting evidence implicating CSC in the initiation and progression of dysplastic oral epithelium to squamous cell carcinoma (SCC), the molecular mechanisms underlying these synergistic biological processes remain unclear. This review will examine the mechanisms that underlie the transformation of normal epithelial stem cells into CSC and the mechanistic link between CSC, TAM, and EC in the growth and the malignant conversation of dysplastic oral epithelium.

## Introduction

Oral SCC is one of the most common malignancies worldwide (1). While there has been a modest decrease in the incidence of this disease, particularly in high-income countries, it continues to be a burden in developing countries. Additionally, in the last decade, there has been an increase in the percentage of young patients developing oropharyngeal SCC due to human papillomavirus (HPV) infection (2–5). The main risk factors for oral cancer are smoking, alcohol consumption, and DNA viruses (e.g., HPV). Unfortunately, efforts to limit the incidence of oral cancer have been met with limited success. A more effective strategy would be identifying the incipient precursor lesions, oral epithelial dysplasia (OED), at risk for malignant transformation (6–8).

While it is well known that dysplasia often precedes the development of oral SCC, the mechanism underlying the progression of epithelial dysplasias to SCC remains uncertain (6–8) CSC and the tumor microenvironment play a critical role in tumor progression. This review will highlight three cell populations that are key to tumor progression: cancer stem cells, vascular endothelium, and macrophages. The mechanism underlying the conversion of epithelial stem cells to cancer stem cells and their role in initiating crosstalk between these cell populations in driving OED to SCC will be the focus of this review.

## What defines a premalignant lesion/condition

The term “premalignant” or “precancerous” is best defined as a lesion or condition that is at increased risk of developing into an SCC (9, 10). There is considerable uncertainty when assessing the malignant potential of premalignant lesions (6). Not all clinical lesions with microscopic alterations associated with premalignancy will progress to SCC (11–13). Some of these lesions will rapidly progress to SCC or have a prolonged latency before progressing to SCC. Still, others may completely regress (6, 8, 11, 13). In addition, premalignant lesions may evolve from benign lesions that exhibit none of the recognized stages of malignant progression (8, 14). Current clinical and histopathologic criteria used to assess the risk of progression of premalignant lesions to SCC are often unable to reliably predict malignant potential (6, 11). In addition, the histologic criteria for evaluating premalignant lesions often vary from examiner to examiner (15–19).

## Disorders associated with increased risk of progression to squamous cell carcinoma

Localized lesions and systemic conditions most commonly associated with an increased risk for progressing to SCC include leukoplakia (white patch), erythroplakia (red patch), proliferative verrucous leukoplakia, lichen planus, oral submucous fibrosis, chronic hyperplastic candidiasis, actinic cheilitis, reverse smoking, betel nut chewing, discoid lupus erythematosus, and the inherited disorders dyskeratosis congenita and Fanconi anemia (6, 19). From a clinical perspective, lesions of most concern often present as leukoplakia with or without a red component. Although these disorders have an increased statistical risk of malignant progression, it is difficult to predict the clinical outcome for any individual lesion. In addition, despite the relative ease of monitoring oral precancerous lesions, there are still no reliable biomarkers that distinguish lesions that will progress to cancer from those that will not.

## Leukoplakia: the precancerous sentinel lesion

Oral leukoplakia is the most common precancerous lesion. It presents as a white patch on the surface of the oral mucosa (8, 20–22). There is some variability in the risk of progression depending on its location in the oral cavity, regional geographic differences, and risk factors (tobacco smoking vs. tobacco chewing). Additionally, what is considered precancer (visual or histopathologic) varies from examiner to examiner (16). The frequency of malignant transformation of oral precancerous lesions varies with the quality and type of outcome assessed, the type of clinical studies conducted (prospective vs. retrospective), and length of follow-up (12). Also, there is considerable variation in the rate and frequency of progression of oral leukoplakia to invasive oral SCC (8, 16, 20–23). Our current understanding of the mechanism underlying malignant progression of OED remains limited. Several studies have investigated potential predictive markers of progression. The most common of these has been p53, proliferation markers Ki67 and PCNA, and a limited number of other molecular biomarkers, including cell cycle proteins, loss of heterozygosity (LOH), a range of cell surface and stromal proteins, and aberrant signaling pathways (24). To date, no single or combination of biomarkers has fulfilled the promise of predicting the onset of SCC at its earliest stages. Despite these limitations, important new information about the role of CSC and the tumor microenvironment in cancer initiation and progression has shed some light on the mechanism underlying the progression of precancerous lesions.

## Precancerous oral epithelium evolves from carcinogen-initiated cancer stem cells

It is generally agreed that adult stem cells are the targets of carcinogenic agents (25, 26). There are four stages to the carcinogenic process. These include initiation, promotion, malignant transformation, and progression (27, 28). For cancer to develop, stem cells must first be exposed to an “initiating agent.” Initiation occurs following exposure to a chemical or physical carcinogen or an infectious agent. Initiation induces a reversable epigenetic modification of DNA or an irreversible molecular lesion that makes the initiated cell susceptible to the growth-promoting effect of a promoting agent (25, 26, 29, 30). Clinical and laboratory studies suggest that carcinogenesis is a process that requires multiple exposure to promoting agents (25, 31–33). Normal stem cells become immortalized, presumably due to a mutational event involving either a protooncogene or tumor suppressor gene (34–37). Once a cell is initiated, the affected cell may persist for many months or years before the second “promoting” event takes effect (38, 39).

Studies in humans and animals have provided compelling evidence that the initiated cells are *de facto* preneoplastic. Lesions described as leukoplakia that microscopically exhibit severe dysplasia or carcinoma *in situ* have taken an irreversible step toward malignant transformation (38–40). Next the initiated cell undergoes clonal expansion, during which additional changes allow it to express the malignant phenotype (25). This process, operationally, is irreversible. Several animal models have supported this concept (27, 28, 32, 39). This process is followed by transforming initiated stem cells into cancer stem cells, setting the stage for other mutational events that lead to cellular transformation and tumor progression. If malignant transformation is to occur the initiation and promotion events must occur in a specific sequence. This four-stage model of carcinogenesis has endured for over fifty years (27, 39).

Once a stem cell is initiated, it is susceptible to the effects of promoting agents that relieve cells from growth constraints and expand their numbers, escaping cell death leading to an accumulation of abnormal cells (25, 41). Skin papillomas, enzyme-altered liver foci, breast nodules, colon polyps, and dysplastic oral leukoplakia harbor initiated cells that have undergone clonal expansion following exposure to a promoting agent (28, 38). Promoting agents are, for the most part, not mutagenic. Therefore, they do not induce the formation of malignant tumors. Instead, they increase the frequency of genetic and epigenetic mutations that position the abnormal cells to undergo malignant conversion. Promoting agents include hormones such as estrogen, the drug diethylstilbestrol, and various chemicals such as per- and polyfluoroalkyl substances (PFAS). Initiation and promotion must occur in sequence. When the initiation and promotion processes are reversed, i.e., when cell are first exposed to a promoting agent followed by an initiation event, cells will not undergo malignant transformation (38, 40). Animal models of skin and liver cancer have shown that initiated cells must be exposed to promoting agents repeatedly before malignant transformation results (25, 42).

Malignant conversion is when precancerous cells express the malignant phenotype. This process requires the accumulation of mutations. Malignant transformation occurs after repeated exposure to a promoter. The frequency of exposure needed to cause malignant conversion varies from tissue to tissue, but the malignant phenotype is eventually expressed. Tumor promotion contributes to the process of carcinogenesis by expanding a population of initiated cells at increased risk for malignant conversion. The relatively low probability of malignant transformation can be increased substantially by repeated exposure of precancerous cells to DNA-damaging agents. This process is mediated through activation protooncogenes and inactivation of tumor suppressor genes (34–36, 43, 44).

**Figure 1:**
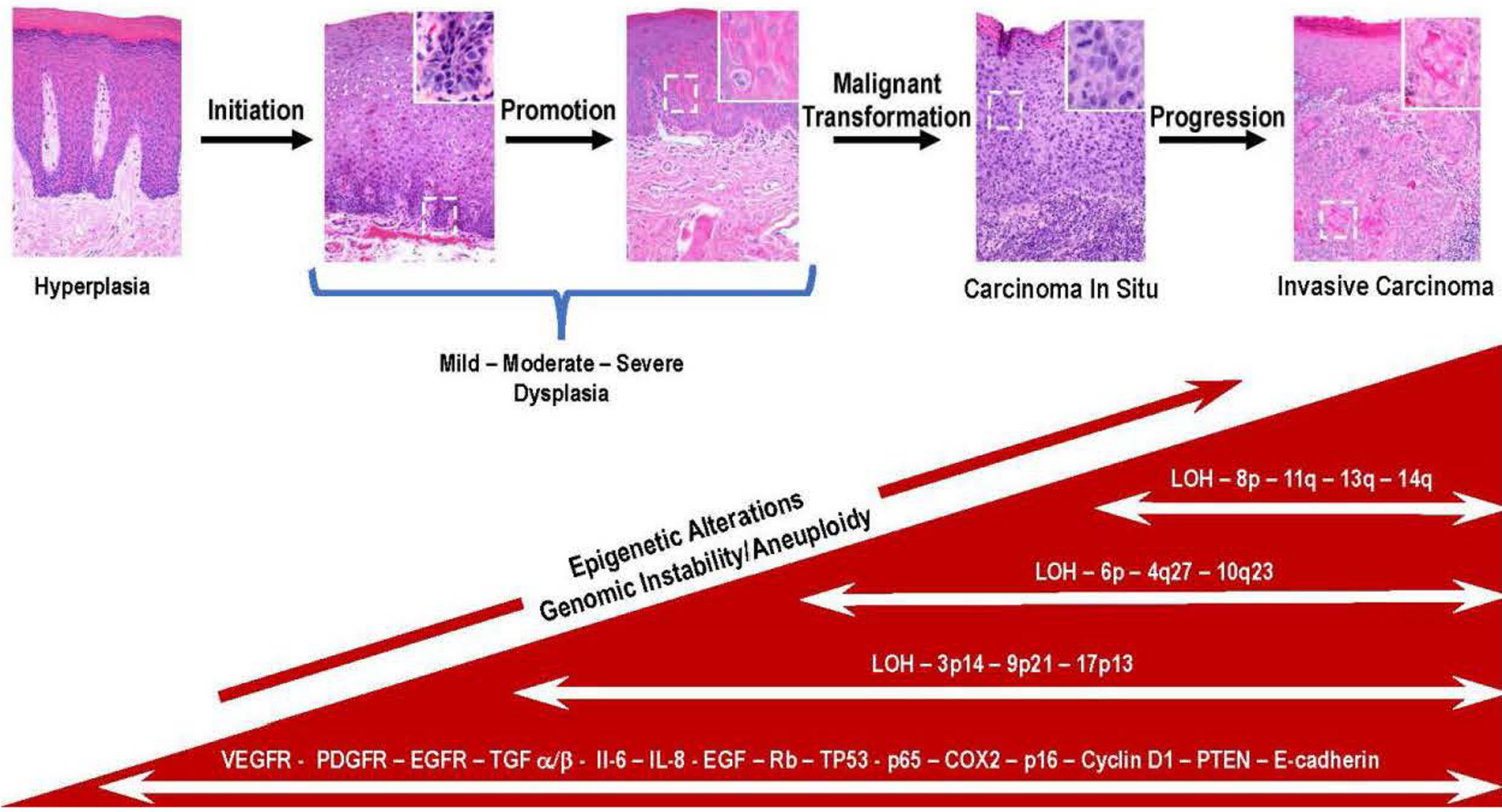
Key genetic and molecular events during the progression of premalignant oral epithelium to oral squamous cell carcinoma are depicted in this multistep model of histologic progression. The histological sequence of events seen in the progression of premalignant epithelium is initiated in the basal cell layer following exposure of stem cells to carcinogens. This results in irreversible damage and or epigenetic modification of DNA resulting in the emergence of a population of cancer stem cells. The subsequent exposure to promoting agents contribute to the clonal expansion of initiated cancer stem cells. With the continued exposure to promoting agents, a subset of initiated stem cells undergoes malignant conversion (i.e., carcinoma *in situ*). Over time cancer stem cells and bulk tumor cells acquire additional mutations many which involve loss or inactivation of tumor suppressor genes (TP53, PTEN, LOH) and overexpression of molecular mediators (Il-6, IL-8, VEGFR, PDGF). The bidirectional exchange of information between cancer and host cells contribute to acquisition of more aggressive phenotypes by the invading cancer cells (100X magnification, inset, white boarder, 400X).

Once dysplastic cells have undergone malignant conversion, they enter the progression phase where they acquire more aggressive characteristics over time. A prominent feature of the malignant phenotype is increased genomic instability and unregulated growth. Further genetic and epigenetic changes occur during this process, including activating additional protooncogenes and the functional loss of tumor suppressor genes. These genetic alterations confer a growth advantage upon cells and increase their capacity for invasion and metastasis. The determining factor is the accumulation of these mutations, not the order or the stage of tumor development (25). Figure 1 depicts the relationship between the process of carcinogenesis with the progression of premalignant leukoplakia to SCC.

## Cancer stem cells sustain tumor growth and progression

Cancer stem cells (CSC) comprise a small subpopulation of cancer cells with the unique capacity to sustain tumor growth and drive tumor progression. CSC are primarily responsible for the failure of conventional and precision therapies (45–48). The properties of stemness, self-renewal, multipotency, and differentiating into heterogeneous cancer cell types define the CSC that make up the tumor mass (49–51). CSC are phenotypically and functionally heterogeneous. The phenotypic diversity gives rise to increased numbers of tumor-initiating cells (52–54). CSC have been identified in most human cancers, including breast, brain, and oral and head and neck SCC (55–58). CSC often reside at the advancing front of invasive SCC, where they are surrounded by their non-stem cell progeny and a variety of host cells that confer a survival advantage on CSC (47, 59, 60). The majority of CSC reside within a 100-mm radius of the perivascular niche (61). The perivascular niche serves as a biological nursery that helps CSC maintain their stem cell phenotype (59, 61). The perivascular niche provides the soil for CSC self-renewal and maintenance, stimulating essential signaling pathways in CSC and leading to the secretion of factors that promote angiogenesis and long-term growth of CSC (47, 62). The essential nature of the perivascular niche was demonstrated by Krishnamurthy et al. who showed that selective ablation of microvessels that comprise the perivascular niche resulted in the reduction in the population of CSC (61).

Recent studies have revealed the indispensable role of the IL-6 signaling in facilitating the acquisition of cancer stem cell functions in coordination with NF-kB-dependent inflammatory signals derived from tumor cells and host cells (63–65). IL-6 is a pro-inflammatory cytokine that activates JAK/STAT3 pathway (66–68). IL-6 levels have been shown to correlate with tumor progression in a number of cancer types including oral and head and neck cancer (69–71). In addition, IL-6 is a predictive marker for recurrence rate and overall survival of head and neck SCC patients (72, 73). Studies have shown that tumor cells acquire metastatic potential through IL-6/STAT3 pathway(74). IL-6 is secreted by many different cells, including T and B cells, monocytes and macrophages, endothelial cells, fibroblasts, and CSC (47, 61). Endothelial cells secrete high levels of IL-6 and expression of IL-6R or its co-receptor gp130 at the invasive tumor front is strongly correlated with poor patient survival (47, 61). Furthermore, endothelial cell-secreted IL-6 induces epithelial mesenchymal transition and enhances migration in head and neck CSC (61). Collectively, these results demonstrate that endothelial cell-secreted IL-6 induces a migratory phenotype in head and neck CSC. It has been suggested that therapeutic blockage of the IL-6 pathway might prevent and/or delay progression of oral and head and neck SCC (47).

Several surface markers, such as CD47, CD44, CD133, and Musashi-1, a stem cell marker fond in several tissues including oral squamous epithelium, are expressed by head and neck and oral CSC along with cytoplasmic enzymes such as aldehyde dehydrogenase (ALDH) (45, 47, 61, 75, 76). CSC, together with host cells are responsible for driving tumor progression. CSC are also responsible for local recurrence, metastatic spread, and therapeutic failures (77, 78). Studies have demonstrated that initiated CSC are present in dysplastic oral epithelium (79, 80). Progression of oral cavity cancer appears to be associated with the increased presence of CSC (79, 80). CD44 is a well- studied markers of CSC in oral cancer (81, 82). It has been shown that the presence of CD44 positive cells in patients is strongly correlated with the progression of premalignant lesions to a more aggressive stage of development (severe dysplasia and carcinoma *in situ*). This is further evidence in support of the idea that CSC play a role in the progression of dysplasia to invasive SCC (80).

## Cancer stem cells, vascular endothelium, and tumor-associated macrophages: an ill-fated relationship that amplifies tumor progression

CSC exist within a complex microenvironment. Bidirectional communication between CSC and host cells plays an essential role in augmenting the tumor-promoting functions of CSC. Recent studies suggest that bidirectional communication between vascular endothelial cells, macrophages, and CSC is central in orchestrating tumor progression (83, 84).

Vascular endothelial cells and macrophages extend the tumor promoting effect of CSC in several ways (83–85). In addition to their role in establishing a stem cell niche, endothelial cells provide essential nutrients to a rapidly growing population of tumor cells through their ability to amplify the blood supply to tumors on demand(86–89). Neovascular endothelial cells and CSC have a coregulatory function(85, 90). Several differentially expressed genes in endothelial cells that are usually quiescent are upregulated during angiogenesis (86, 91). These include, among others, growth factors and growth factor receptors, matrix metalloproteinases, inhibitors of angiogenesis, and NF-kB-regulated signaling pathways (85). In addition, endothelial cells produced cytokines, including HGF, PDGF, PIGF, and IL-6, that stimulate the self-renewal and enhances the survival of CSC (92, 93).

Besides their role in self-renewal CSC from a variety of tumors have been directly implicated in activating the angiogenic switch (85, 86, 91). For example, CSC from gliomas and ovarian cancer produce elevated levels of proangiogenic mediators (85, 90). Tissue samples from patients with glioblastoma multiforme as well as CSC isolated from these tumors revealed high levels of the angiogenic mediators VEGF, VEGFR1 and VEGFR2 and the hypoxia-inducing agents HIF1*α*, HIF2*α*. Many of these proangiogenic mediators ae also produced by cells within the tumor microenvironment that communicate with CSC to promote angiogenesis (93).

It is well established that tumor angiogenesis plays a central role in tumor progression. In many cancers, tumor-associated endothelial cells have a higher proliferative rate as compared to the established microvasculature where endothelial cells are normally quiescent (94). It is well documented that the increased microvascular density (MVD) that accompanies tumor development has shown prognostic value in a variety of cancers and is an established indicator of tumor progression (80, 95, 96). A study comparing low risk (mild dysplasia) to high risk (moderate and severe) mucosal lesions showed a statistically significant correlation with MVD expression. Also increasing numbers of CD44^+^ CSC and CD31^+^ endothelial cells was positively correlated with increased MVD and high-risk dysplasias progressing to SCC (80, 97). These results further support the notion that CSC and tumor-associated endothelial cells have biologically synergistic roles in tumor progression. Furthermore, epithelial dysplasias that undergo malignant transformation are positively associated with CSC enrichment. This observation further confirms the role of vascular niche, endothelial cells, and CSC in tumor progression (80).

Tumor-associated macrophages (TAM) are a heterogeneous population of cells. In addition to phagocytic and antigen-presenting functions, they play an essential role in inflammation, resolving infections, and tissue repair. In contrast, macrophages are also responsible for tissue damage, chronic inflammatory diseases, autoimmune disorders, and tumor growth and progression (98, 99). This array of divergent functions is due in part to their ability to undergo polarization into two phenotypically distinct subpopulations designated as M1 and M2 macrophages (98–100). M1 macrophages produce an array of proinflammatory cytokines and reactive oxygen molecules that promote Th1-mediated tumoricidal responses. The M1 polarization in macrophages is mainly regulated by distinct transcriptional networks consisting the Notch, NF-*κ*B, TGF-*β*, Wnt/*β*-catenin, and MAPK (101–105). On the other hand, M2 macrophages are considered anti-inflammatory, are involved in tissue remodeling and immune tolerance, and have protumor functions that facilitate tumor progression. Transcriptional control in M2 is mediated through STAT1 and STAT2 activation in response to type-1 *α* and *β* interferons (106). Additionally, STAT isoforms, including STAT3 and STAT6, modulate M2 polarization (104, 107–114).

The process of tumor progression is frequently associated with a phenotypic switch from M1 to M2 in tumor-associated macrophages (115). The polarization of macrophages is a labile process where the proportion of M1 and M2 TAM with tumors will vary depending on the functional status of tumor microenvironments (116, 117). Through distinct sets of autocrine and paracrine signaling molecules, transcription factors, and epigenetic modifiers, tumor cells can further differentiate TAM into subsets of tumor-promoting macrophages capable of altering the genetic and phenotypic profiles of tumor cells (118, 119).

**Figure 2:**
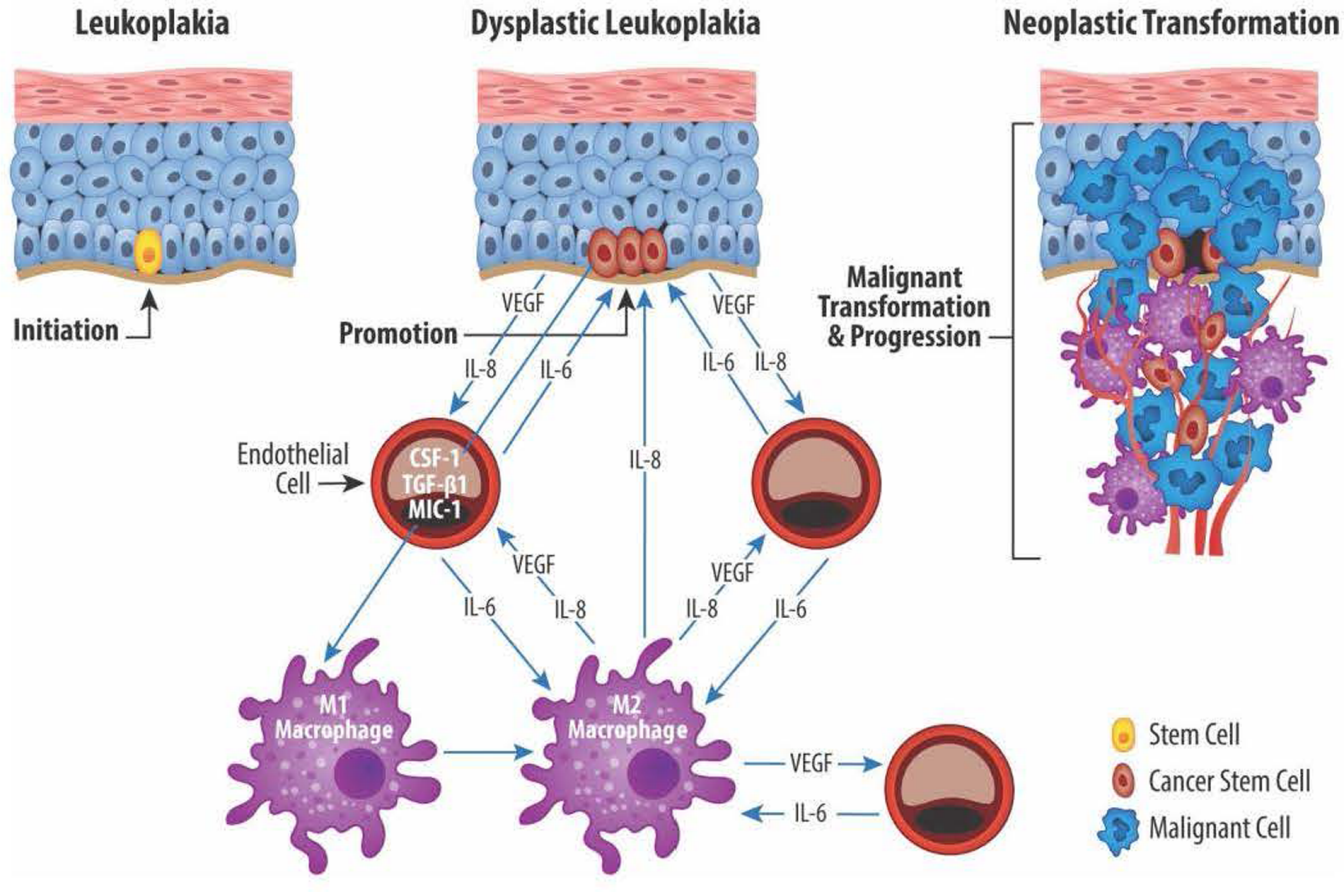
Reciprocal exchange of cytokine mediators between cancer stem cells (CSC), endothelial cells (EC), and macrophages (M1 and M2). CSC located in the basal layer of dysplastic epithelium produce, among others, the angiogenic mediators VEGF and IL-8. EC in turn produce IL-6 which plays an important role in maintaining the CSC stemness. EC also play a role in transitioning proinflammatory M1 macrophages to protumor M2 macrophages *via* production of IL-6, colony stimulating factor 1 (CSF-1), TGF-*β*1, macrophage inhibitory cytokine 1 (MIC-1). Lastly, M2 macrophages promote angiogenesis *via* production of VEGF and IL-8 and help maintain CSC stemness through IL-8 production.

Macrophages and endothelial cells influence the CSC functions through a series of reciprocal interactions between CSC, endothelial cells, and TAM (101). Neovascular endothelial cells and M1/M2 TAM produce mediators that enable CSC to maintain their stem cell phenotype while maintaining TAM proangiogenic and protumor functions (120). Cytokines produced by endothelial cells and TAM stimulate the self-renewal and survival of adjacent CSC (121, 122). The proximity of vascular niche endothelial cells to CSC provides the nurturing environment necessary to maintain the CSC phenotype and enable CSC self-renewal and long-term survival. Together, the complementary networks created by cancer stem cells, endothelial cells, and TAM contribute to tumor proliferation, growth, invasion, metastatic activity, and treatment failures. Figure 2 shows the reciprocal relationships between CSC, endothelial cells, and TAM and the mediators responsible for the protumor effects that define this relationship.

## Discussion

The tumor microenvironment is defined by the distribution of various stromal cells and their sequential and mutually beneficial cellular interactions. Recent studies of CSC and the tumor microenvironment have revealed novel insights into the complex mechanisms that drive tumor progression and underlie treatment failures. For example, the bidirectional flow of growth factors and cytokines between CSC and host cells (EC, and TAM) promotes TAM protumor functions, sustains angiogenesis, maintains the CSC phenotype, thereby facilitating tumor growth and progression (123). CSC, like their normal stem cell counterparts, are phenotypically and functionally diverse. Studies of various human tumors have shown that phenotypically diverse subsets of CSC are present within tumors. However, their relationship with one another and their role in tumor development and progressions remain unclear. Changes in the tumor microenvironment, such as the degree of hypoxia, the composition of the inflammatory infiltrate, and their cytokine mediators, undoubtedly play a crucial role in determining the types of CSC cells that populate a tumor.

It is well established that cancer evolves from a series of random mutational events that lead to a complex series of aberrant cellular, genetic, and molecular processes. The ability of cancer cells to adapt to changes in their environment provides them with a selective growth and survival advantage. By co-opting the regenerative properties of the host, cancer cells can utilize the bidirectional flow of information between cancer cells and host cells to their advantage. It is this unique ability of cancer cells to thrive in an otherwise hostile host environment that characterizes the dynamic cancer ecosystem (124–127). Continued exploration of the mechanisms that underlie the physiologic reprogramming of CSC and the signals involved in orchestrating CSC diversity will be necessary to design therapeutic strategies that target CSC and its progeny at the incipient stages of tumor development.
